# A rare association of neuromyelitis optica, antisynthetase, and antiphospholipid syndrome

**DOI:** 10.1002/ccr3.7873

**Published:** 2023-10-30

**Authors:** Dóra Nemes‐Tömöri, Richárd Csabalik, Edit Boglárka Nagy, Tibor Béldi, Gyöngyike Emese Majai

**Affiliations:** ^1^ Department of Clinical Immunology University of Debrecen, Internal Medicine Debrecen Hungary; ^2^ University of Debrecen, Neurology Clinic Debrecen Hungary; ^3^ University of Debrecen, Medical Imaging Clinic Debrecen Hungary

**Keywords:** antiphospholipid syndrome, antisynthetase syndrome, Devic's syndrome, neuromyelitis optica

## Abstract

The association of neuromyelitis optica concurrently with two other autoimmune diseases is rare. Neuromyelitis optica should be taken into consideration when evaluating the symptoms of the patient as a differential diagnostic aspect.

## Graphical abstract

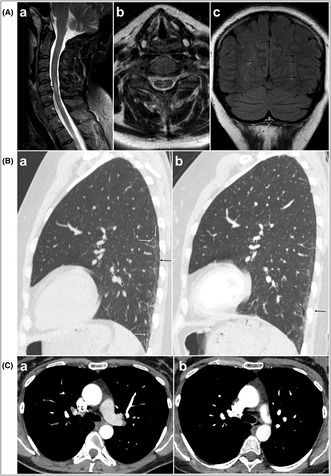

## 1 INTRODUCTION

Neuromyelitis optica (NMO) is a demyelinating autoimmune disease often associated with IgG1 antibodies specific to the water channel protein aquaporin‐4 (AQP‐4) that can be found on astrocytes. These autoantibodies are able to penetrate the blood‐brain barrier, connecting to AQP‐4 leading to the activation of the complement system, causing complement‐dependent cytotoxicity. By inducing natural killer cells antibody‐dependent cytotoxicity causes astrocyte damage.^1^ Taking into consideration the AQP‐4‐antibody serostatus, NMO can be divided into two subgroups: AQP‐4 IgG seropositive which means the presence of antibody against AQP‐4 and seronegative meaning the absence of it.^2^ In AQP‐4 IgG seronegative neuromyelitis optica spectrum disorders (NMOSD), different pathologies were detected. In these cases, the target is myelin oligodendrocyte glycoprotein (MOG), a myelin protein expressed on the myelin sheath. Autoantibodies against MOG lead to myelin loss. There are several case reports in the literature presenting Sjögren's syndrome and systemic lupus erythematosus associated with NMO.^3^ Less often other autoimmune diseases such as rheumatoid arthritis, systemic sclerosis, or antiphospholipid syndrome (APS) can also occur together with NMO. However, co‐occurrence of antisynthetase syndrome (ASS) and NMO was reported only once.^4^ Here we report the case of a female patient with antisynthetase syndrome, secondary antiphospholipid syndrome, and NMO.

## 2 CASE REPORT

**FIGURE 1:**
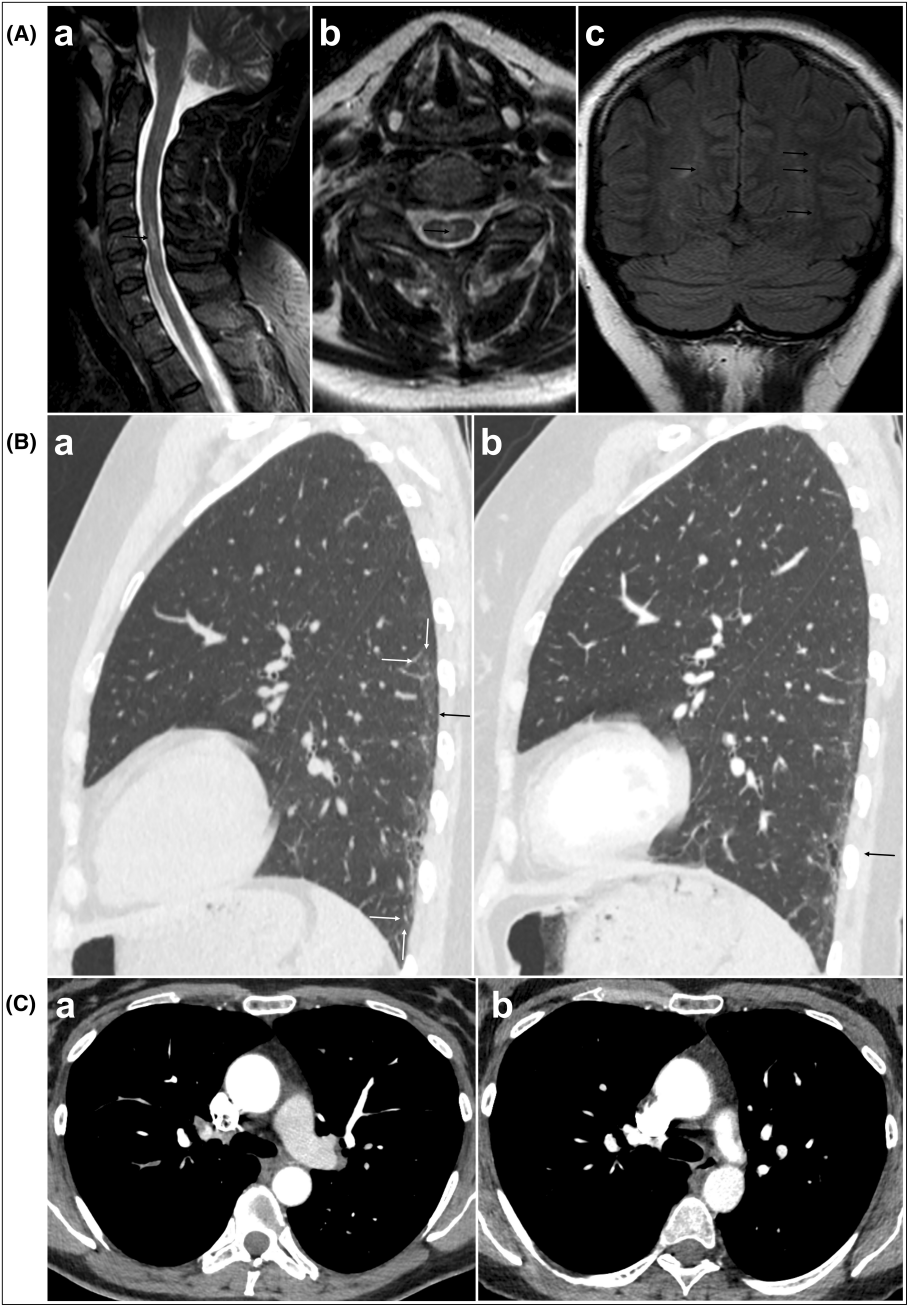
(A) Central nervous system involvement in neuromyelitis optica. (a) Cervical spine T2‐weighted sagittal and (b) axial MRI image shows short transverse myelitis with high T2 signal (arrows). (c) Brain coronal fluid attenuated inversion recovery (FLAIR) image shows some demyelinating lesions with high signal. (B) Nonspecific interstitial pneumonia (NSIP) pattern. (a) Sagittal CT image shows ground glass opacities with basal peripheral predominance (between white arrows) and subleural sparing (black arrow). (b) Three months later, the extent of the ground glass opacities is minimally reduced. Arrows indicate traction bronchiectasis. (C) Acute pulmonary thromboembolism. (a) Axial contrast‐enhanced CT scan shows embolus in a segmental pulmonary artery of the right upper lobe (arrow). (b) Three months later the lumen was recanalized.

We present the case of a 50‐year‐old female patient who was admitted to our department for the first time 5 years ago with the symptoms of erythema on the face, fatigue, and eye‐mouth dryness, with the history of Raynaud's phenomenon. Previous laboratory investigations revealed anti‐β2‐glycoprotein I IgG positivity without thrombotic event. Familial history was positive to thrombosis and prostate cancer. In her gynecological history, she was pregnant and gave birth two times without any complications. She was controlled regularly at our outpatient clinic; however, these visits have been stopped. In the spring of 2021, she returned with expanded range of symptoms including rash on the chest (V sign), effort dyspnoea, discrete joint pain, burning pain in the left lower limb, and an electric shock like sensation in the right upper limb occurring during the anteflexion of the neck (Lhermitte sign). Previous laboratory tests revealed fluctuating leukopenia but normal level at the time of presentation, anti‐SSA, anti‐SSB, anti‐β2‐glycoprotein I IgG, and anti‐Jo1 positivity (Table 1). Sjögren's syndrome was excluded by negative Schirmer test and sialometry. Due to lower limb hypesthesia and present Lhermitte sign magnetic resonance imaging (MRI) was performed two times. Hyperintense lesions on T2‐weighted and FLAIR sequences were present both times, the optic nerve was not affected. The first cranial MRI data suggested cerebral vasculitis. Digital subtraction angiography was performed with negative result. Spinal cord MRI revealed the involvement of the spinal cord, meaning mostly central lesions localized to the cervical and thoracic segments (Figure 1A/a–c). Suspecting NMO liquor examination was performed, that showed anti‐AQP4 positivity. Based on the neurological symptoms, the presence of anti‐AQP4 antibody and the involvement at least three vertebral segments the diagnosis of NMO could be established.

**TABLE 1:** Immunoserological findings throughout the years.

Autoantibodies	Years					Reference range
	2017	2019	2021	2022	2023	
ANF titer	1:1280	1:1280	1:2560	1:2560	1:1280	1:80
Anti‐dsDNS	7,8	9,5	n.e.	8	n.e.	0–20 U/mL
Anti‐Sm	<10	<10	<10	<10	<10	0–10 U/mL
Anti‐Sm/RNP	<10	<10	<10	<10	<10	0–10 U/mL
Anti‐SSA	>100	75	89	79	58	0–10 U/mL
Anti‐SSB	66,3	32	33	18	11	0–10 U/mL
Anti‐Jo1	Positive	Positive	Positive	Strongly positive	Strongly positive	
Anti‐β2‐GP I IgG	121,1	171,2	105	108,7	143,7	0–20 U/mL

Abbreviation: n.e., not evaluable.

Further investigations excluded optic neuritis via visual evoked potential. Considering the effort, dyspnoea, anti‐Jo1 positivity, antiphospholipid antibody positivity, spirometry, DLCO (diffusing capacity of the lung for carbon monoxide), and contrast‐enhanced HRCT (high‐resolution computed tomography) were performed showing the pattern of nonspecific interstitial pneumonia (Figure 1B/a,b) and pulmonary embolism (Figure 1C/a,b) in the right upper lobe. Duplex ultrasound confirmed deep vein thrombosis in the right calf. Summarizing all the clinical manifestations (Raynaud's phenomenon, arthralgia, V sign, interstitial lung disease (ILD), deep vein thrombosis, and pulmonary embolism) and the positive immunoserology, antisynthetase syndrome was diagnosed with APS and NMO. Due to the concurrent autoimmune diseases, antisynthetase syndrome, NMO and APS, bolus steroid was administered, and introduction of azathioprine was suggested. As progression of the neurological symptoms appeared and clinically manifested ILD was also present after consultation with neurologist, cyclophosphamide and later one rituximab was started, since rituximab is an off‐label treatment for ILD and NMO.^5^, ^6^ On rituximab and steroid treatment, the neurological symptoms improved, but the ILD showed slight progression suggesting the need of combined immunosuppressive treatment. Considering ILD and the good results of mycophenolate mofetil (MMF) in other autoimmune disease‐associated ILD, MMF was introduced.

## 3 DISCUSSION

Antisynthetase syndrome is a rare autoimmune disorder, a subtype of idiopathic inflammatory myopathies. It is characterized by Raynaud's phenomenon, arthritis, myositis, and ILD. The concomitant occurrence as an initial manifestation of all typical clinical features is uncommon, following the patients with plausible ASS is important for proper diagnosis. In ASS, antibodies against aminoacyl transfer RNA is a typical serological feature, the most common is anti‐Jo1, which is an anti‐histidyl‐tRNA synthetase. Patients with anti‐Jo1 positivity are prone to develop interstitial lung disease.^7^ Antiphospholipid syndrome often occurs secondary as an overlap syndrome to other autoimmune diseases. The typical immunoserological abnormalities include the presence of lupus anticoagulant, anticardiolipin and/or anti‐beta‐2‐glycoprotein I antibodies at moderate or high titer. Repetitive and unexplained deep vein or arterial thrombosis, pulmonary embolism, fetal or early pregnancy loss, placental insufficiency, or preeclampsia are the main clinical manifestations.^8^ In overlap syndromes, the appearing symptoms should be evaluated expansively, as in the presented case dyspnoea might be the sign of ILD or pulmonary embolism, but with one well‐aimed imaging technique both conditions were diagnosed. Weakness of the limbs as a typical symptom of idiopathic inflammatory myopathies is also possible to occur in neuromyelitis optica indicating the involvement of the spinal cord and extra‐CNS manifestations.^9^ Finding the origin of the symptoms is crucial for choosing the most appropriate treatment. In ASS, the treatment is based on the most dominant manifestation. In ASS‐ILD, glucocorticoids are chosen as initial treatment. As maintenance therapy, azathioprine, mycophenolate mofetil, or cyclophosphamide should be considered.^5^ In NMO, intravenous glucocorticoids are the first therapeutic option.^6^ B cells play an important role in the pathogenesis of ILD because of autoantibodies and IL‐6 secreted by B cells. IL‐6 induces pulmonary inflammation and fibrosis.^10^ In the pathogenesis of NMO, B cells and IL‐6 are also found. In NMO, the recruitment of autoreactive B cells and the production of anti‐AQP4 are stimulated by IL‐6.^6^ Monoclonal antibodies against the participants of the pathogenesis in both diseases are available off‐label. Rituximab, which is an anti‐CD20 monoclonal antibody, and tocilizumab, an anti‐IL‐6 receptor antibody, are therapeutic approaches to slow down or stop progression of ASS and NMO.^5^, ^6^

## 4 CONCLUSION

Patients with systemic autoimmune disorders are prone to develop overlap diseases. Re‐evaluating the known and new symptoms are crucial to diagnose the underlying conditions. Neuromyelitis optica, as a differential diagnostic aspect and overlap disease, should be always considered.

## AUTHOR CONTRIBUTIONS

**Dóra Nemes‐Tömöri:** Writing – original draft; writing – review and editing. **Richard Csabalik:** Writing – review and editing. **Edit Boglárka Nagy:** Writing – review and editing. **Tibor Béldi:** Writing – review and editing. **Gyöngyike Emese Majai:** Writing – review and editing.

## FUNDING INFORMATION

This research received no specific grant from any funding agency in the public, commercial, or not‐for‐profit sectors.

## CONFLICT OF INTEREST STATEMENT

Authors have no conflict of interest to declare.

## CONSENT

Written informed consent was obtained from the patient for publication of this case report and accompanying images.

## Data Availability

The authors confirm that the data supporting the findings of this study are available within the article.
